# Artificial Intelligence Aided Design of Tissue Engineering Scaffolds Employing Virtual Tomography and 3D Convolutional Neural Networks

**DOI:** 10.3390/ma14185278

**Published:** 2021-09-14

**Authors:** María Dolores Bermejillo Barrera, Francisco Franco-Martínez, Andrés Díaz Lantada

**Affiliations:** 1 ETSI de Telecomunicación, Universidad Politécnica de Madrid, Av. Complutense 30, 28040 Madrid, Spain; mariadolores.bermejillo.barrera@alumnos.upm.es; 2 Mechanical Engineering Department, ETSI Industriales, Universidad Politécnica de Madrid, Calle José Gutiérrez Abascal 2, 28006 Madrid, Spain; francisco.franco@upm.es

**Keywords:** artificial intelligence (AI), machine learning (ML), tissue engineering, tissue engineering scaffolds, 3D convolutional neural networks (3D CNNs)

## Abstract

Design requirements for different mechanical metamaterials, porous constructions and lattice structures, employed as tissue engineering scaffolds, lead to multi-objective optimizations, due to the complex mechanical features of the biological tissues and structures they should mimic. In some cases, the use of conventional design and simulation methods for designing such tissue engineering scaffolds cannot be applied because of geometrical complexity, manufacturing defects or large aspect ratios leading to numerical mismatches. Artificial intelligence (AI) in general, and machine learning (ML) methods in particular, are already finding applications in tissue engineering and they can prove transformative resources for supporting designers in the field of regenerative medicine. In this study, the use of 3D convolutional neural networks (3D CNNs), trained using digital tomographies obtained from the CAD models, is validated as a powerful resource for predicting the mechanical properties of innovative scaffolds. The presented AI-aided or ML-aided design strategy is believed as an innovative approach in area of tissue engineering scaffolds, and of mechanical metamaterials in general. This strategy may lead to several applications beyond the tissue engineering field, as we analyze in the discussion and future proposals sections of the research study.

## 1. Introduction

Artificial intelligence (AI) and machine learning (ML) methods are reshaping data management, product design, materials science and mechanical engineering, among other industrially and socially relevant fields. As regards AI-aided discovery of materials, the Materials Genome Initiative [1,2,3] stands out as one of the pioneering large-scale projects, which has inspired other technological breakthroughs in innovative materials for advanced industrial applications [4,5]. Usually, AI and ML have been applied to the prediction of final properties and performance of materials from the chemical composition of the bulk materials under study [6,7,8]. In some cases, mechanical properties have been also predicted with ML techniques [9,10]. More recently, our team has focused on the forecasting of tribological properties of hierarchical topographies, advancing in the AI-aided design of textured surfaces and materials [11]. Besides, the progressive application of AI to the prediction, design and control of mechanical properties is already making an impact in the growing family of mechanical metamaterials [12,13], whose application fields include transport, energy, space and health, to cite a few.

Authors consider similar holistic approaches, aimed at promoting accelerated materials development through an intensive use of AI, if further research focusing on materials microstructures and mechanical properties can prove transformative towards high performance materials and devices in several industries.

The biomedical area can greatly benefit from innovative hierarchical mechanical metamaterials, whose multi-scale porous and lattice structures are essential for mimicking the biomechanical properties of human tissues and organs. This recapitulation of the mechanical properties of the cell microenvironment, through implanted artificial materials, constructs or “scaffolds”, helps the cells of damaged tissues receive the adequate mechanical stimuli and is one of the essential strategies in tissue engineering and regenerative medicine [14,15].

The design of successful tissue engineering scaffolds is a challenging engineering task, due to the complexity of the original tissues they aim to repair and the intricate connections between designed geometries, bulk materials and manufacturing tools. FEM simulations are commonly applied to CAD models of tissue engineering scaffolds, so as to analyze their prospective mechanical properties, usually focusing on their stiffness, as a parameter to optimize for enhanced biomimetic behavior [16,17]. However, there are many occasions in which FEM simulations cannot be applied to scaffolding structures or do not adequately predict final performance, due to geometrical complexity, large aspect ratios, or presence of manufacturing defects. Therefore, the use of AI and ML can prove valuable for deciphering this complexity of scaffolding structures and thus supporting bioinspired design approaches.

To mention some examples, AI and ML have been applied to the tissue engineering field for different purposes, in general related with the enhanced design of scaffolds: the ML-guided 3D printing of scaffolding geometries for minimizing defects has been reported [18], to predict vascularization in repair strategies [19] and to correlate in vitro performance with physico-chemical properties [20]. Conventional artificial neural networks are the most used methods for these AI-ML strategies. Nevertheless, the prediction of scaffolds’ mechanical performance directly from the CAD models, especially for cases in which FEM cannot be applied, may require advanced ML tools capable of using more complete descriptions of these complex geometries as input, and not just representative parameters. Quite recently, 2D convolutional neural networks (CNNs), with images as input, have been effectively applied to predicting multiple properties of porous materials, which constitutes a fundamental advance [21].

In the authors’ view, the use of 3D CNNs can constitute a novel and alternative (or complementary) method for predicting the properties of complex porous materials and structures, especially the mechanical properties of tissue engineering scaffolds. Taking into account the success of deep CNNs or 3D CNNs using medical images (i.e., computed tomography-CT-) as input for diagnostic purposes [22,23,24], similar ML strategies and schemes may apply to materials science and engineering.

Our rationale is as follows: the computed tomography of physical objects generates layered 2D images representing the whole geometry of a real object, proving useful as input for 3D CNNs for diagnostic purposes. Then, the use of digital tomography to obtain layered 2D images of virtual CAD models, like the CAD models of tissue engineering scaffolds, may be used as input for successfully predicting, in silico, the properties of the designed geometries.

To demonstrate our driving hypothesis, in this study, we create and characterize a collection of tissue engineering scaffolds and employ digital tomography to obtain the layered 2D images defining the geometries of the scaffolds of the collection. The 3D CNNs are trained using the layered images as input and the characterized properties, namely Young’s modulus, shear modulus and porosity, as outputs. Once trained and validated, employing a varied set of training and validation strategies, the predicting potential of the obtained 3D CNNs is tested with a new set of designs of tissue engineering scaffolds. To rapidly generate the tomographies from the virtual models, we apply a 3D printing slicer software in an innovative way, using the digital masks as the actual layered images that recreate the 3D geometry of each construct. The performance achieved, both in terms of predicting ability, computational speed and global cost, is remarkable.

To the authors’ best knowledge this study represents the first application of 3D CNNs to the AI-aided design of tissue engineering scaffolds. It is also an innovative approach in the area of mechanical metamaterials and may lead to a wide set of applications beyond the tissue engineering realm, as we analyze in the discussion and future proposals sections of the study. The following section describes the materials and methods employed, before dealing with the presentation and discussion of research results. Finally, the more relevant pending challenges, as well as some proposed and expected research directions, are also presented.

## 2. Materials and Methods

### 2.1. Creating a Library of Tissue Engineering Scaffolds with Well-Known Properties

**Figure 1:**
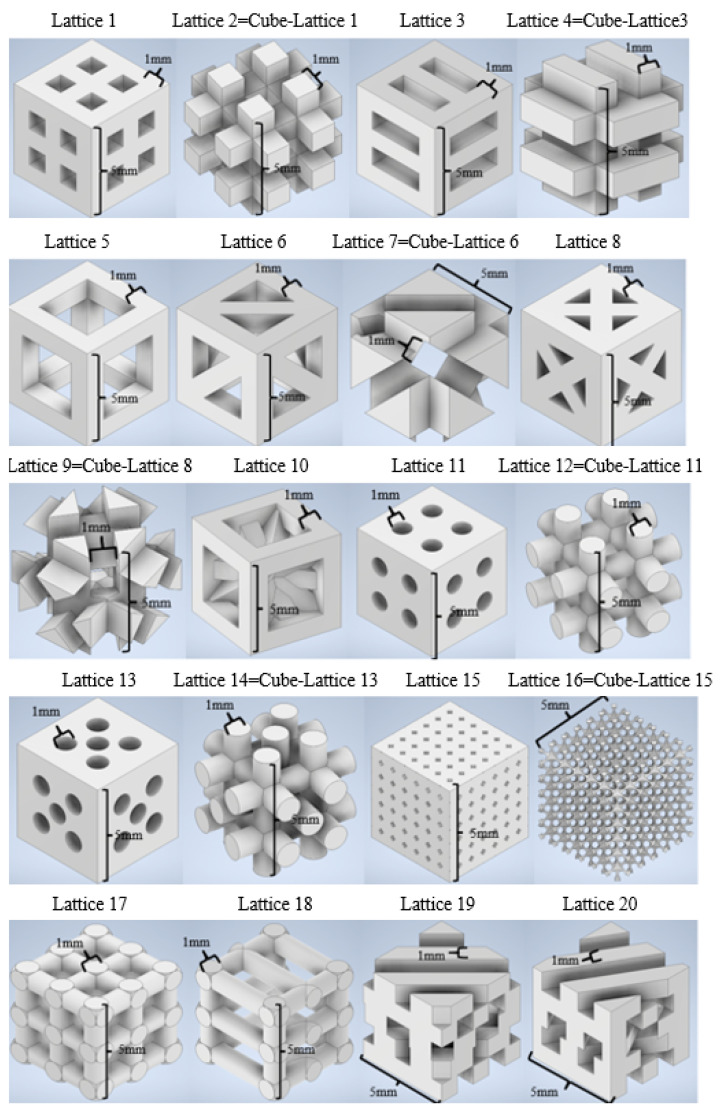
CAD library of tissue engineering scaffolds or mechanical metamaterials. Cell units of 5 × 5 × 5 mm^3^ are shown. Some geometries are obtained by direct Boolean operation, subtracting an already designed lattice to a cube of material.

Computer-aided design and finite element simulations are performed with the support of Autodesk Inventor 2021 (academic license). A collection of 20 lattices or scaffolding geometries is created by means of solid- and matrix-based operations and using Boolean tools. Geometrical diversity is sought, for which some designs have periodic features and remarkable symmetry, while others are conceived as irregular interconnections of trusses to increase the desired diversity. Some lattices are obtained by subtraction of already designed scaffolds to a bulk cubic geometry. In any case, all designed geometries can be considered tissue engineering scaffolds for different tissue repairs, as they resemble the common geometries used for 3D printed scaffolds for regenerative medicine. The cell units of the 20 designed scaffolds are shown in Figure 1, which summarizes the collection of CAD geometries. All of them are inscribed in a cube of 5 × 5 × 5 mm^3^.

Once designed, different properties of the scaffolding units are obtained. Porosity, defined as % of void within the 5 × 5 × 5 mm^3^ working volume, is directly measured with the CAD software. Two main mechanical properties are obtained for each lattice, the compression modulus and the shear modulus. The compression modulus of an elastic material is defined as the ratio between applied stress and resulting strain when that material is under compression. The shear modulus is defined as the ratio between applied shear stress and resulting shear strain. Due to the varied mechanical stimuli that tissue engineering scaffolds suffer in service, both properties are interesting from a biomechanical point of view.

The mechanical characterization of the different lattices, for obtaining the compression and shear moduli, is done in silico, using the FEM simulation capabilities of the used software. Lattices are meshed using tetrahedral elements of 0.05 mm. ABS, as conventional thermoplastic, is employed as bulk material. A normal or transversal distributed load of 25 N is applied, leading to an equivalent normal or shear stress considering that the lattices occupy a section of 5 × 5 mm^2^. As boundary condition, each lattice is fixed on the face opposite to the face where the force is applied. Once simulated, the equivalent compression or shear moduli are obtained. Dividing them by the actual compression or shear moduli of the bulk material used for the simulation (ABS) eliminates the influence of the raw material, leading to relative values only dependent on the actual lattice geometry, which are typically used for comparing mechanical metamaterials and in some materials selection strategies and in Ashby’s diagrams.

The results from in silico characterization for the different lattices are included in Table 1 and subsequently used for training the 3D CNNs. For training purposes, such values are normalized or scaled to the [0, 1] range, as this leads to better and faster 3D CNN convergence. For further processing, the CAD models of the designed lattices or unit cells are stored as binary .stl (standard tessellation language or stereolithography) files with a mesh precision of 0.05 mm, which proves adequate for employing Chitubox as slicing software for the digital tomographs, as explained further on.

### 2.2. From 3D CAD Files to Digital Tomographies as Input for 3D CNNs

Chitubox v.1.8.1 basic (Chitubox, Zhongcheng Future Industrial Park, Hangcheng Avenue, Baoan District, Shenzhen, Guangdong, China 518128) is a free 3D printing software designed to edit and slice 3D CAD models. It also provides tools for CAD transformation including rotating, scaling, mirroring, repairing, hollowing, cloning, etc. With the help of Chitubox, the designed lattices are sliced, transforming their 3D geometry into a set of black and white images that resemble the layered images of CT-scans or MR imaging. A resolution of 1440 × 1440 pixels per slice is chosen and a distance of 0.25 mm between slices, along z-axis, is selected. Each slice is a cut of a scaffold, with a section of 5 mm^2^, and generates an image of 1440 × 1440 pixels. Thus, each pixel has a lateral size of 5 mm/1440. This leads to a set of 20 images per lattice capable of representing the 3D geometries with a remarkable level of detail, at least similar to the level of detail used when actually printing similar tissue engineering scaffolds.

**Figure 2:**
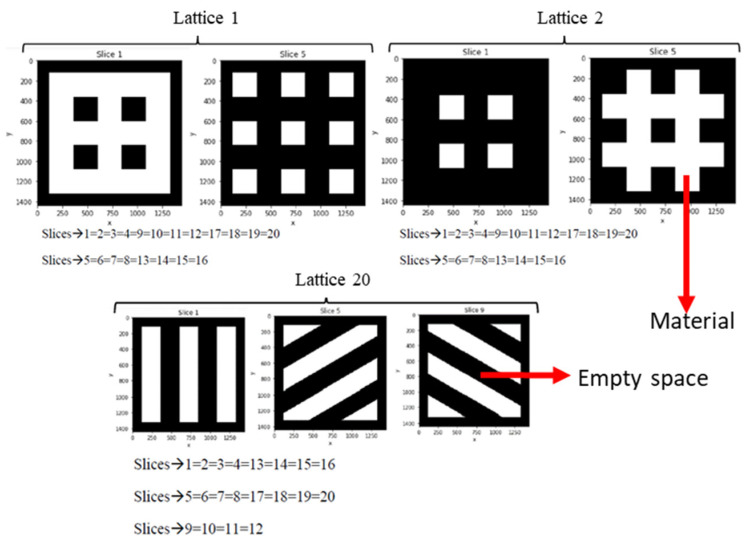
Examples of digital tomographies, for different 3D CAD models of the designed lattices, achieved employing a lithographic slicer for 3D printing. Results for lattices 1, 2 and 20 (see Figure 1) are presented by means of example. Each lattice is transformed into 20 slices, many of which are coincident due to the periodic nature of the lattices.

Figure 2 provides examples of these digital tomographies, for different 3D CAD models of the designed lattices, achieved employing Chitubox as lithographic slicer for 3D printing. Results for lattices 1, 2 and 20 (from Figure 1) are presented in Figure 2 by means of example. Each lattice is transformed into 20 slices, many of which are coincident due to the periodic nature of these lattices. In these images, as happens in real CT-scans and in the Hounsfield scale utilized for the dicom (digital communications in medicine) standard, black represents empty spaces or voids and white corresponds to the actual scaffolding material. These sets of images are used as input for the 3D CNNs, for describing the geometries of the different lattices, while the in silico obtained properties (porosity, compression and shear moduli) are used as output, for training and validating purposes, as detailed in the following subsection.

The approach resembles pioneering experiences of 3D CNNs in medicine, but employing digital slices of CAD models, instead of real CT images.

### 2.3. Structuring and Training 3D CNNs for Predicting Mechanical Properties

For the development of the artificial intelligence/machine learning model, the Python programming language in version 3.8.5 (Phyton Software Foundation) is employed. This is the most used language in machine and deep learning since it is open source and provides the necessary tools to carry out this type of process effectively and relatively easily, thanks to available powerful dedicated libraries. Besides, Python interpreter allows to run programs written in Python language [25]. Different libraries for data analysis, data processing and deep learning are also employed, whose main features and application purposes are described below:

NumPy version 1.19.2: specialized in numerical calculation and data analysis for large volumes of data. This library incorporates matrices (arrays) that allow to represent data collections of the same type in several dimensions. It also incorporates very efficient functions for manipulating arrays [26]. This library is fundamental in study since a 3D CNN understands a 3D image as a 3D array.

Matplotlib version 3.3.4: develops quality 2D and 3D graphics with a few lines of code, uses static, animated and interactive figures, allows to take full control of line styles, axis properties, among other options [27]. In short, this library allows the visualization of data and results.

Scikit-learn version 0.24.1: main machine learning library in Python, providing different tools for predictive data analysis and calculation of metrics, such as mean square error [28], needed in supervised learning. It includes generalized linear regression models.

Scikit-image version 0.18.1: dedicated to image processing, it allows reading and displaying images from a file, binarizing, resizing, segmenting images, adjusting their contrast and color and other typical image processing operations [29].

TensorFlow version 2.3.0: compiles and trains artificial intelligence models with ease, using intuitive and high-level application programming interfaces (APIs), such as Keras, with immediate execution and allowing immediate model iteration and easy debugging [30].

Keras version 2.4.0: part of the TensorFlow library from TensorFlow version 2.0.0 and it has a more friendly code. It covers every step of the deep learning workflow, from data management, training, configuration and evaluation of the model to obtaining predictions or testing the artificial intelligence model. This makes it a widely used deep learning framework, whose guidelines are public [31].

For the installation of all the previous libraries, a Python distribution called Anaconda is used, which already includes the Python interpreter installed and the Numpy and Matplotlib libraries used. The other libraries are installed thanks to the Anaconda package manager called conda. In this case, the package manager is conda version 4.10.1.

Anaconda, in addition to the package manager, includes a desktop application called Anaconda Navigator, which allows to manage packages, as well as run applications such as the Jupyter Notebook development environment. In this case, Anaconda Navigator has Jupyter Notebook version 6.3.0 and it is the development environment used.

Once the software and libraries are installed, data are preprocessed and augmented, the structure for the 3D CNNs are defined. Finally, training, validation and testing strategies are designed and implemented.

Since each slice has very large dimensions (1440 × 1440 pixels), images are resized to 32 × 32 pixels using Scikit-image library again. Although the resolution is lower, the network is able to understand patterns and learns faster during training. Then, considering that 3D CNNs understand images as arrays, the 20 slices of each lattice are concatenated along a new axis, in this case the z axis. A total 20 3D arrays of 0 s and 1 s are obtained that represent the 20 CAD cellular scaffolds. The Numpy library is used for this operation.

**Figure 3:**
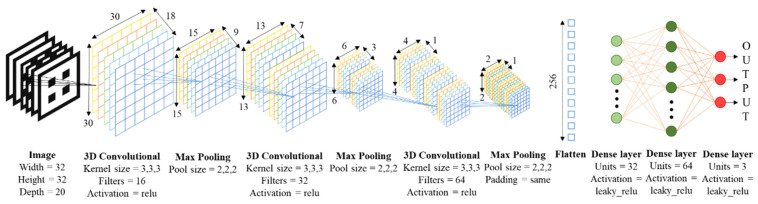
Proposed structure for the 3D CNNs: from 3Dslices models to useful performance properties. Block diagram, structure and details of convolutional neural network.

Regarding the architecture for the 3D CNNs, we opt for a structure involving input images (representative of the 3D geometries), convolutional and max pooling layers and fully connected dense layers leading to the outputs (porosity and mechanical properties), as schematically presented in Figure 3.

Summarizing, the design of 3D CNNs’ architectures and their training and validation is carried out thanks to Keras library. To analyze which is the best model of all those designed, the following strategy is carried out. A total 70% of the 3D structures without any transformation are used as training data and 30% of them as validation. Regarding the loss function and the metric, the selected model is the one whose mean square error in training and validation is closer to 0 and the lowest. Moreover, if it is possible the validation MSE should be slightly lower than that of training. This means that the convolutional neural network “learns” from the training data and can generalize to data outside of that set. Therefore, the cross-validation method is used to estimate the precision of the different models. Data segregation, from the total training data set into training and validation data, is done using Scikit-learn library. The selected model consists of a 3D convolutional layer with 16 filters each with dimensions 3 × 3 × 3 pixels. A Relu activation function is used, and filters weights are applied randomly.

Subsequently, a 3D max pooling layer with a filter dimension of 2 × 2 × 2 pixels is placed. After this layer, a batch normalization layer is used and then a dropout layer with an index equal to 0.3. The batch normalization layer normalizes and scales its inputs by applying a transformation that keeps the mean output close to 0 and the output standard deviation close to 1 [32]. Standardizing the activations of the previous layer means that the subsequent layer makes about the propagation and distribution of the inputs during the weight update will not change dramatically. This has the effect of stabilizing and accelerating the deep neural network training process [33].

The dropout layer randomly sets the input units to 0 with a rate of speed at each step during the training time, which helps prevent overfitting. The inputs that are not set to 0 are scaled by 1/(1-rate) so that the sum of all the inputs does not change [34]. A dropout layer supposes the unlearning of the neural network, which prevents the network from learning in excess from the training data, “memorizing them”, and not being able to predict any new input data (problem well-known as overfitting). This succession of layers is then repeated, but this time the convolutional layer uses 32 random filters. Then it is repeated again, but the convolutional layer uses 64 random filters and the max pooling layer maintains the last dimension of the data it receives to avoid obtaining a negative dimension. Otherwise, there would be no dimensions to apply the max pooling layer. The combination of the three package of layers described adopts the shape of bottleneck, because the greater the depth of the convolutional neural network, the greater the abstraction. To extract all the feature information the deeper is the layer, smaller are the filters used per convolution layer and it is often used with a larger amount of filters. Finally, a flatten layer is placed, a succession of dense layers with 32 and 64 neurons respectively, and another dense layer with 3 neurons because the network predicts three variables (provides 3 outputs) from each input (lattice geometry as slices). All dense layers use a Leaky Relu activation function. Main features of CNN are seen in Figure 3, where the output shape of the layers is shown around the different blocks of convolution and the flatten layer.

**Figure 4:**
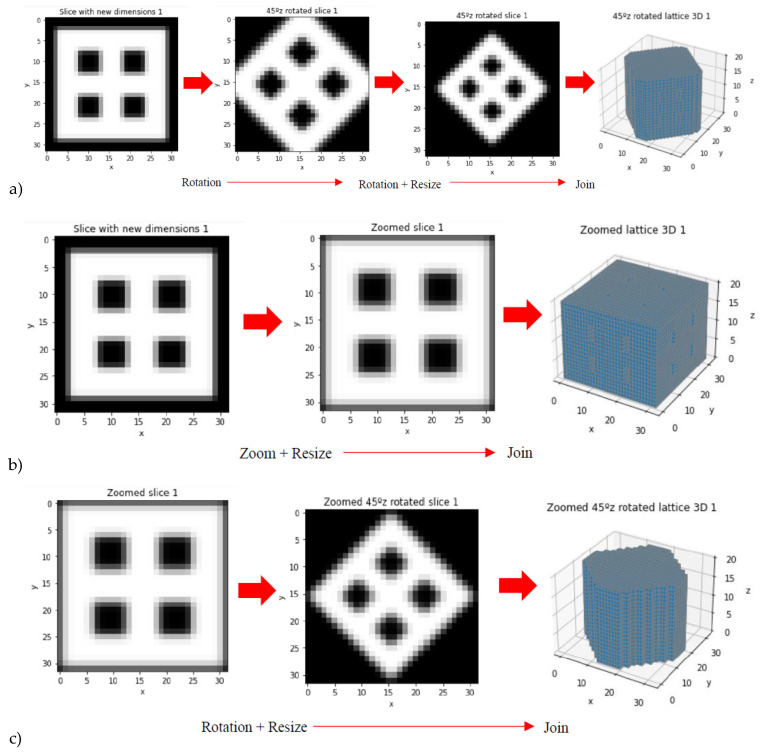

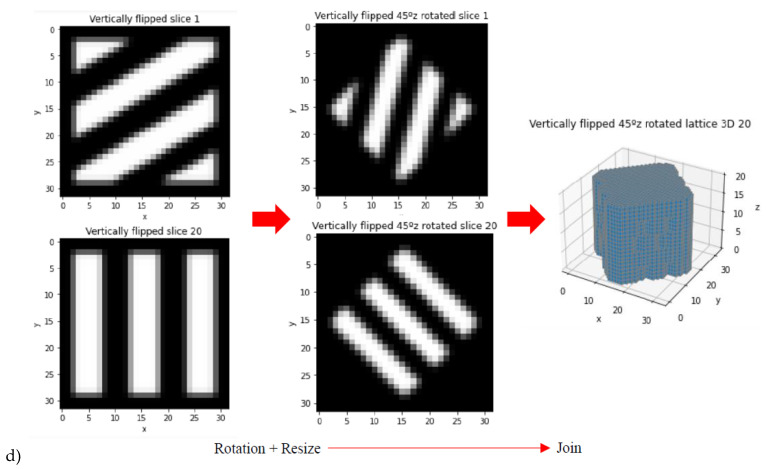
Examples from data augmentation strategies: (**a**) Rotation around z axis and resizing. (**b**) Zoom. (**c**) Zoom, rotation around z axis and resizing. (**d**) Vertical flip and rotations around z axis.

Taking into account that a library of 20 scaffolding lattices may be limited as training and validation set for AI/ML strategies, data augmentation is performed. Such data expansion is achieved through rotations around z-axis, zooms and resizing, vertical flips, rotations around x- and y-axes, to cite some options used. Different examples of these data augmentation strategies are shown in Figure 4 by means of example and summarized in Table 2.

The CNN shown above is designed based on two main examples [35,36]. The structure is adapted to solve the question dealt in this paper, using an iterative process to find a good performance. With the selected model, six training and validation strategies are carried out. The six strategies consist of expanding the training data and modifying the validation data randomly as summarized in Table 2 below.

**Table 2:** Summary of the six training and validation strategies employed.

Strategy	Number of Lattices Used	Number of Lattices Used for Training	Number of Lattices Used for Validation	Data Augmentation Strategy
1st strategy	20	14 random ones	6 random ones	-
2nd strategy	120	114 random ones	6 random ones	Rotations around z-axis (15°, 30°, 45°, 60°, 75°)
3rd strategy	240	234 random ones	6 random ones	Previous rotations plus zoomed-in lattices
4th strategy	360	354 random ones	6 random ones	Previous rotations and zooms (240 lattices) plus vertical flips without zoom (120 lattices more)
5th strategy	480	474 random ones	6 random ones	Addition of horizontal flips (120 lattices more)
6th strategy	680	674 random ones	6 random ones	Addition of rotated initial lattices around x- and y-axes (15°, 30°, 45°, 60°, 75°, 200 lattices more)

### 2.4. Testing and Validation of the Global Strategy

**Figure 5:**
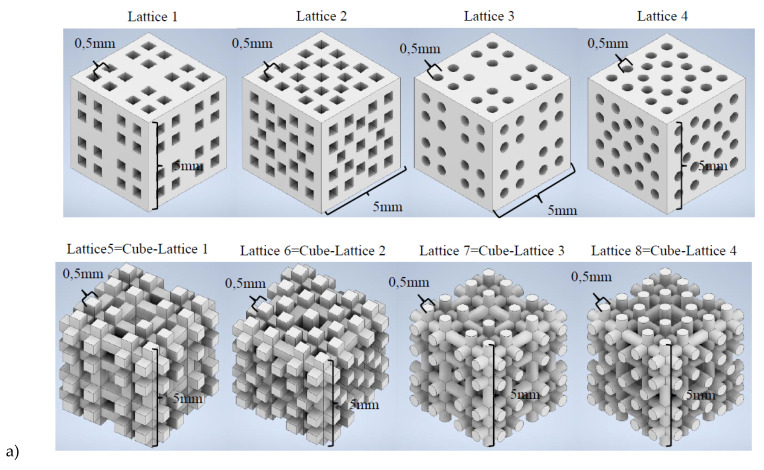

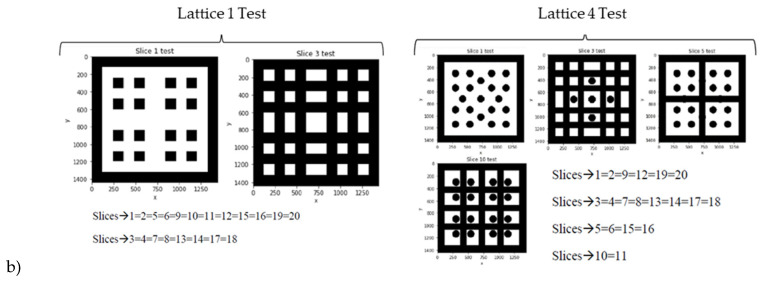
Testing samples for validating the 3D CNNs performance. Eight new lattices are designed, and their properties are simulated, with the support of FEM simulations as reference, and predicted with the previously trained and validated 3D CNNs. (**a**) Computer-aided designs. (**b**) Digital tomographies as input to the 3D CNNs (examples for lattices 1 & 4).

Once trained, the 3D CNNs are tested, and their predicting ability validated, in a real use case scenario. For this purpose, a new library of scaffolding geometries with 8 lattices (see Figure 5a), completely different from those included in the initial collection of 20, is designed. These lattices are sliced (see examples from Figure 5b), following the procedure described in Section 2.2 and the different 3D CNNs are employed to predict their properties. In parallel, the methods from Section 2.1 are applied to in silico characterize these new lattices. The obtained properties (Table 3) are compared to the predicted ones. The precision of the different 3D CNNs, obtained as detailed in Section 2.3 employing varied training strategies, is analyzed. Results are presented and discussed in the following section.

## 3. Results and Discussion

### 3.1. CAD Models, Digital Tomographies, and Training and Validation of 3D CNNs

The initial CAD library of 20 scaffolding geometries, used for training and validating the 3D CNNs, is already an interesting result, which improves with the addition of the eight additional new scaffolds of Figure 5a, designed for testing the global AI/ML strategy. This designed collection of microstructured geometries is a starting point, aimed at creating the most comprehensive library of tissue engineering scaffolds with information about their biomechanical performance, which can be continuously updated. Such updates can be used for further training the 3D CNNs, once additional designs, simulation and testing results upon CAD files or physical prototypes are available. The scaffolds’ library already includes several CAD files in .ipt (Inventor parts) and .stl (standard tessellation language -or stereolithography-) formats, as well as their equivalent slices (or digital tomographies) stored in the form of arrays. The library, and its future additions, are openly available for researchers in the field, wishing to collaborate or test related approaches, linked to the AI- or ML-aided development of tissue engineering scaffolds and metamaterials.

Among other remarkable results of the study, authors would like to highlight the possibility of recapitulating the three-dimensional geometries of complex CAD objects by using a 3D printing slicer, Chitubox in this case, and employing the obtained slices as input for 3D CNNs. Examples of the application of the slicer are shown in Figure 2 for the initial training library and in Figure 5b for the new lattices used for testing the global AI-ML strategy. The use of digital tomographies is demonstrated useful for training, validating and testing deep convolutional neural networks, as the employment of images from CT scans had already proven highly useful for the progressive application of AI/ML methods in diagnostic medicine. Although recent studies have also combined machine learning and FEM simulations to predict the mechanical properties of biomaterials lattices and biomechanical structures [37,38], they have normally relied on conventional artificial neural networks (ANNs) with a few parameters as inputs/outputs that describe slight variations in thickness, size, length or density. Moreover, 3D CNNs, loaded with digital slices, receive the whole geometry as input and outperform simpler ANNs, especially when the geometrical complexity increases and when the diversity of geometrical inputs does not allow for a parametrization. It is also well-known that CNNs achieve better results in image processing than ANNs because CNNs preserve and analyze the sequence of data. These types of algorithms recognize the position and relationship between near pixels of the inputs.

**Figure 6:**
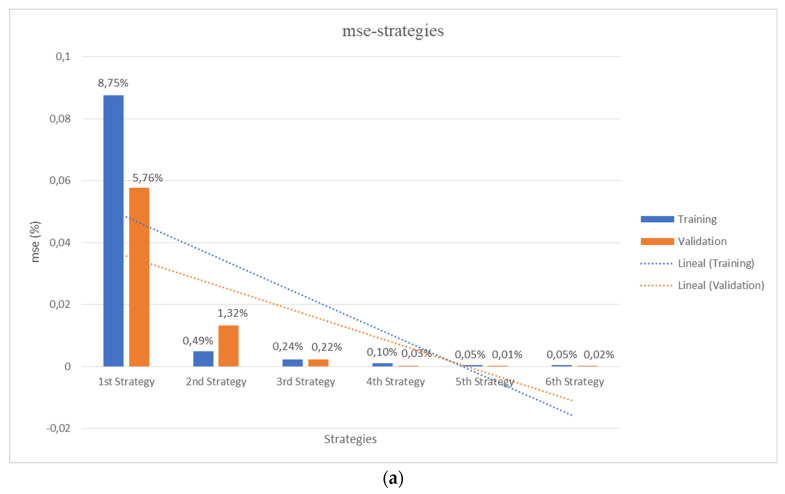

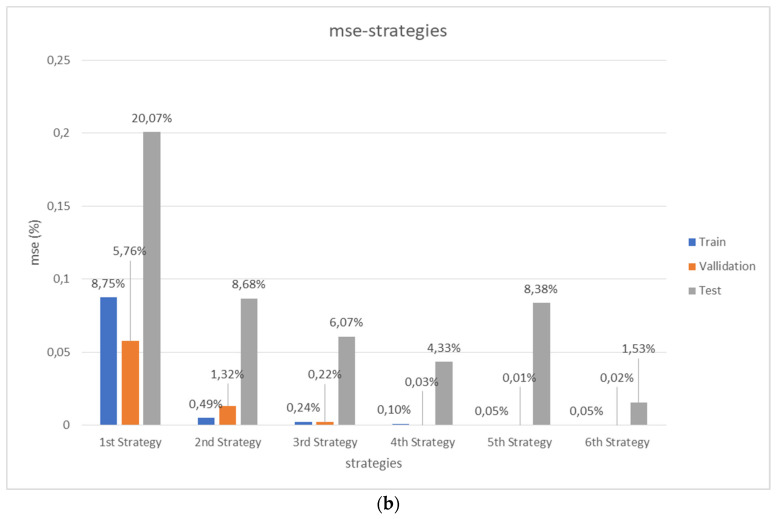
Comparative performance of the different strategies. (**a**) Results from 3D CNNs training and validation according to the 6 detailed strategies with increasing number of input lattices (see Section 2.3). (**b**) Final performance, comparing the testing errors with those from previous training and validation processes for the different trained and validated 3D CNNs. Mean square errors (MSE) in % are presented.

Arguably, CT scans or magnetic resonance (MR) images carried out upon physical samples, materials, products and real patients, may synergize with the use of digital tomographies, like those obtained using 3D printing slicers upon CAD files, for fostering the application of AI/ML methods in a wide set of scientific-technological disciplines, from materials science and engineering to regenerative medicine. The training and validation work properly, the initial library of scaffolds and the errors decreasing with the addition of lattices to the training set, as seen in Figure 6a, thanks to the strategies for data augmentation described in Section 2.3.

The performance of the trained 3D CNNs in a real-life scenario, for predicting the mechanical properties of new lattices added to the library, is discussed in the following subsection.

### 3.2. Performance of the Structured and Trained 3D CNNs: Predictions vs. Real Performance

Once trained, the 3D CNNs are employed to predict the porosity and mechanical performance of the new design lattices of Figure 5, whose characteristic properties are shown in Table 3. The new lattices are sliced, following the same procedures applied to the original library of 20 samples, and evaluated with the available 3D CNNs that provide different porosities, compression and shear moduli as outputs. The predicted outputs are compared with values from Table 3. This corresponds to a real-life scenario, in which the trained networks face completely new geometries, never used previously for training or validation, and process them to predict their properties for classification or selection purposes.

Figure 6 schematically presents a comparative overview of the performance of the different strategies. On the one hand, Figure 6a shows the results from 3D CNNs training and validation according to the six detailed strategies with progressively increasing number of input lattices (as already described in Section 2.3). On the other hand, Figure 6b shows the final performance, comparing the testing errors with those from previous training and validation processes for the different trained and validated 3D CNNs. In all cases, the mean square errors (MSE, in %) are presented. Additional level of detail, for the different structures and strategies, is in the Appendix A, which presents a complete report of the characterized and predicted values for the different properties of the new lattice collection used for the final testing of the global strategy, according to the explanation provided in Section 2.4.

In agreement with the initial expectations, the training and validation errors rapidly decay with the increase of input/output data. Besides, such decreasing trend is also seen for the testing experiment, although the fifth strategy gives an unexpectedly high error for the testing set, possibly due to over-fitting or over-learning during the training of such specific 3D CNNs with the fifth strategy. In any case, although the first 3D CNN trained and validated with a set of 20 samples leads to a high prediction error for the testing set of 20% (MSE), the 3D CNNs trained with increasing number of lattices lead to MSE values of c.a. 9%, 6%, 4% and 1.5% for strategies 2, 3, 4 and 6 respectively. It is important to remark that the MSEs values are calculated on scaled output.

These results are quite remarkable and are in the common range achievable by FEM simulations upon CAD files or by mechanical testing upon manufactured samples. When these results are compared with those from FEM simulations, it is important to note that when geometries are too complex or involve multi-scale features, FEM simulations are sometimes impractical or extremely demanding in terms of computational resources and simulation time. However, for the described 3D CNNs, the computational times required for training and validating are c.a. 0 s, 3 s, 6 s, 9 s, 13 s and 18 s for strategies 1 to 6. Once implemented, the actual testing or prediction of porosity and mechanical properties of new samples is almost immediate.

Regarding precision of slicing and number of images employed as input, for converting the 3D geometries into a collection of sliced images, to have a sort of “digital tomography”, we employ a 3D printing slicer for photopolymerization systems (Chitubox), as has been explained. These types of slicers normally cut the CAD models according to the z-axis resolution of printing machines, which typically print with vertical steps ranging from 50 to 300 microns. We considered that a digital tomography, using a separation between slices of 250 microns, which provides 20 images for each 5 × 5 × 5 mm^3^ scaffold, would be adequate for illustrating the methodology. It is a common value, both in the 3D printing field and in the materials science field when performing tomographies of porous materials, as well as in medicine when exploring patients. A higher precision in the z-axis would arguably lead to an increased precision, but it is also true that the geometric features of the CAD models are in many cases periodic, and their details are normally larger than 500 microns, which even with 20 images per geometry leads to many repeated slices in each sample. We consider that the selected resolution in the z axis is an adequate compromise between precision and processing speed.

In terms of compromise between precision and speed, due to the fact that each geometry was represented by 20 images of 1440 × 1440 pixels, which led to a large collection of high-quality images, resizing of slices to 32 × 32 pixels is applied. We understood that resolution would be lower, as previously mentioned, but for processing purposes this resizing is interesting, as it reduces the number of pixels by a factor above 2000. We believe that this is a common practice and we have followed examples from previous studies and a library that provides this possibility. In the end, results show that the network can detect the patterns, somehow understanding that a higher presence of white pixels is linked to more dense and rigid structures, although precision could probably be improved by using higher quality images for training and much larger processing times.

Furthermore, the results show an exciting point about the use of zoom for data augmentation. Before evaluating the results, the authors assumed that the zoom could probably adversely affect the performance of 3D CNN. Quite the opposite, the use of zoom improves the performance of the AI demonstrating the neural network appreciate the relation between empty and material space (black and white pixels) in each image, and their connections with density and stiffness (whiter structures tend to be denser and stiffer). The improvement using zoom is slight, but it can be appreciated when comparing the errors between 2th and 3th strategies, where the unique difference is the use of data augmentation by zoom-in. This technique allows the networks to learn from where an image is taken (closer or further away) that is, the networks learn different features due to the relationships between different pixels, their positioning, their colors and not by the fact of moving away or approaching.

Interestingly, although physical properties like Young’s modulus are not scale independent and although authors used the zoom as an option for data augmentation, but without initial high expectations about its potential benefits, its employment has shown some benefits. We explain this considering that the zoom may help the network to predict some phenomena like stress concentration, in which the details of the connections between trusses play a relevant role, even if further studies are still needed to analyze this possibility and the potential generalization problems that using a zoom and other data augmentation strategies might generate.

The metric mean absolute error (MAE) for the strategies is included in Table 4. MAE would provide a fast and easy outlook about the performance in the different scenarios. As it can be seen in Figure 6, the analysis of the MSE, MAE metrics revealed the increased power for predicting the output when 3D CNN is trained with more varied data, thanks to the use of data augmentation. The 6th strategy outperforms the others in all outputs with a global MAE of 7.057. The strategy global MAE is calculated as the average of the MAE for the three outputs and presented in more detail in the Appendix A.

## 4. Challenges and Future Proposals

### 4.1. Potentials, Limitations, and Challenges of the Study

AI and ML techniques have intrinsic limits, including the need of large data for achieving desired results, the “black box” problem, issues with over fitting, interpolative nature, making them work adequately only for data with similar features to those used for training, among others, which we have tried to avoid along our research. However, such common AI and ML drawbacks may lead to the failure of similar learning strategies to those applied here, once translated to other problems in the tissue engineering field or in connection with the prediction of properties for mechanical metamaterials.

Although the data set used started with a library of just 20 geometries, the employment of varied data augmentation processes proved useful for minimizing errors and leading to acceptable results, despite the considerable overfitting to the validation sets perceived. Considering the preliminary nature of the study, which aims at validating an innovative strategy for the AI-aided design of tissue scaffolds, we believe that the study may be of interest for researchers in the field and for progressing towards the AI-aided design of other porous materials and metamaterials. In any case, for solving related problems by applying similar methods, it would be important to count with larger data sets or to expand them even more for enhancing results.

In spite of the intrinsic limitations of AI and ML techniques in general, and of the 3D CNNs employed here in particular, for some applications they may outperform other well established simulation methods. For example, FEM simulations may lead to unaffordable computational costs when evaluating highly complex geometries, especially when aspect ratios are high or when multi-scale “fractal-like” features are present, for which the use of AI methods may prove competitive indeed, if they were adequately trained and validated. Such multi-scale and fractal-like features are common in the tissue engineering field, as the scaffolds are normally designed to mimic the intricate geometries of nature. Apart from the applicability of these tools to biomechanical problems, other studies have also shown the benefits of resorting to deep neural networks when dealing with extremely complex simulations, as a way for achieving an attractive trade-off between cost and accuracy [39].

Ideally, the developed AI and ML tools will lead, not just to predicting porosity and mechanical properties, but also to a better understanding of the behavior of tissue engineering scaffolds and to more adequate AI-aided processes for the engineering of biomaterials for regenerative medicine.

### 4.2. Future Research Proposals

Thinking of future research directions, in the authors’ opinion, it would be interesting to augment the library of scaffolding materials to a larger extent, so as to obtain a truly universal tool for supporting designers of tissue engineering scaffolds (and of mechanical metamaterials in general). This augmentation can benefit from open-source approaches, sharing CAD models, evaluated data and developed and trained networks among the research community. In this way, the effort dedicated to collecting the data can be amortized over many groups collaborating. To progress in such direction, we provide the whole characterized and predicted data of the new lattices in the Appendix A, as a complement to the data shown in Table 1 and Table 3. The CAD models of our collection are also available by email request, with the hope of initiating fruitful collaboration with colleagues.

In the tissue engineering field, many of the complex multi-scale geometries of porous tissue scaffolds are a consequence of more traditional manufacturing processes that do not follow an existing computer-aided design, as happens with 3D printing or additive manufacturing technologies. Such traditional scaffold fabrication processes usually obtain highly random porous structures, with biomimetic features, by phase-separation, solvent casting, gas-assisted injection molding or foaming. In those cases, digital tomographs of CAD files, like those used in this study, are not viable for obtaining the input images and expanding the library. As an alternative, the use of micro-CT or micro-MRI is proposed, measuring upon real manufactured samples and using them as input for increasing the versatility of the library and related 3D CNNs, constitutes another remarkable option.

Towards more holistic scaffold design strategies, apart from considering porosity and compression and shear moduli, other properties of the designed scaffolds may prove fundamental for a successful performance of the implantable construct. Characterizing the geometries of the library considering properties like diffusion coefficient, pressure drop of circulating fluid, natural frequencies of vibration, surface to volume ratio, to cite a few, and verifying the possibility of training the 3D CNNs for predicting them, may lead to a more complete and effective design tool. In addition, a higher degree of versatility is expectable if grayscale images, instead of black and white slices, are employed.

Once improved, automated design procedures may be implemented and the 3D CNNs may be used for selecting the best scaffolds according to a set of properties that should be optimized. This may help, in the future, to reach a tool capable of autonomously designing tissue engineering scaffolds and even mechanical metamaterials in their more general conception, if the presented strategies are applied to other application fields.

## 5. Conclusions

This study has dealt with the application of 3D convolutional neural networks to the prediction of different essential properties of tissue engineering scaffolds. The 3D CNNs have been trained using digital tomographies obtained from the CAD models and results from CAD measurements and FEM simulations. Their predictive performance has been analyzed by using the trained 3D CNNs to forecast the properties of a new set of tissue engineering scaffolds and the results using a collection of networks differently trained has also been discussed. Although the performance is not ideal and additional research efforts are needed, the results obtained validate an AI-based methodology for predicting the properties of complex structures, which may be applicable when the computational cost of other simulation methods results unaffordable. The study has dealt with the biomechanical performance of tissue engineering scaffolds, but similar strategies may be applied to a wide set of properties in the emergent area of metamaterials. Theoretically, these processes can be applied to the automated design or discovery of microstructures with desired mechanical properties, especially if the analyzed current limitations are answered and if multidisciplinary research approaches are promoted.

## Figures and Tables

**Table 1:** Summary of lattices’ properties, characterized employing CAD measurement and FEM tools.

Lattice nº	Relative Porosity (%)	Relative Compression Modulus E_Relative (%)	Relative Shear Modulus G_Relative (%)
Lattice 1	35.2000	39.5401	13.0767
Lattice 2	64.8000	18.5385	3.1629
Lattice 3	48.0000	35.8848	5.9301
Lattice 4	52.0000	30.0935	6.1656
Lattice 5	64.8000	7.9094	2.0723
Lattice 6	40.0864	8.8467	9.8194
Lattice 7	59.9136	8.1076	3.4133
Lattice 8	22.7736	29.9924	19.2402
Lattice 9	77.2264	10.4106	0.9591
Lattice 10	42.8672	21.1969	12.3903
Lattice 11	28.6480	48.9160	18.4019
Lattice 12	71.3520	14.3817	2.1877
Lattice 13	36.9416	25.2379	14.4458
Lattice 14	63.0584	18.2058	2.43572
Lattice 15	6.31440	88.0836	33.2377
Lattice 16	93.6864	1.4064	0.0704
Lattice 17	45.7240	29.0857	11.1030
Lattice 18	57.0864	26.7598	4.6127
Lattice 19	45.3984	10.3383	6.9349
Lattice 20	45.1312	20.6479	7.1886

**Table 3:** Summary of new lattices’ properties, characterized employing CAD measurement and FEM tools.

Lattice nº	Relative Porosity (%)	Relative Compression Modulus E_Relative (%)	Relative Shear Modulus G_Relative (%)
New Lattice 1	35.1376	42.6450	13.7545
New Lattice 2	47.5272	21.5032	8.9878
New Lattice 3	28.6504	52.3093	18.7747
New Lattice 4	38.6408	35.7584	14.7934
New Lattice 5	64.8624	18.4618	3.1262
New Lattice 6	52.4728	25.2521	4.6577
New Lattice 7	71.3496	14.3355	2.1183
New Lattice 8	61.3592	17.7851	1.1427

**Table 4:** Report of mean absolute error. Characterized vs predicted values for the different properties of the new lattices. Please see Appendix A for additional details, metrics and information about different strategies and lattices.

Strategy	Relative Porosity MAE	Relative Elastic Modulus MAE	Relative Shear Modulus MAE	Strategy Global MAE
1st strategy	39.073	26.814	13.193	26.360
2nd strategy	18.476	18.926	9.263	15.555
3rd strategy	17.646	20.207	5.634	14.496
4th strategy	14.577	12.108	6.823	11.169
5th strategy	15.496	18.713	9.289	14.500
6th strategy	7.665	10.264	3.242	7.057

## Data Availability

Data, source code of the developed software and developed 3D CNNs are available upon reasonable request.

## Appendix A

**Table A1:** Porosity, Elastic Modulus and Shear Modulus Mean Absolute Error (MAE) estimation for each training strategy.

Lattice nº	Simulated Relative Porosity (%)	Predicted Relative Porosity (%)	Relative Porosity AE	Relative Porosity MAE	Simulated Rel. Elastic Modulus E_Relative (%)	Predicted Rel. Elastic Modulus E_Relative (%)	Rel. Elastic Modulus E_Relative AE	Rel. Elastic Modulus E_Relative MAE	Simulated Rel. Shear Modulus G_Relative (%)	Predicted Rel. Shear Modulus G_relative (%)	Rel. Shear Modulus G_Relative AE	Rel. Shear Modulus G_Relative MAE
1st strategy												
Lattice 1	35.138	−0.569	35.706	39.073	42.645	63.123	20.478	26.814	13.755	27.629	13.874	13.193
Lattice 2	47.527	−0.750	48.277		21.503	29.878	8.374		8.988	12.163	3.175	
Lattice 3	28.650	−0.559	29.209		52.309	82.533	30.224		18.775	25.614	6.839	
Lattice 4	38.641	−0.780	39.421		35.758	56.540	20.782		14.793	30.589	15.796	
Lattice 5	64.862	25.843	39.019		18.462	−0.074	18.536		3.126	1.489	1.637	
Lattice 6	52.473	−0.764	53.237		25.252	77.633	52.381		4.658	33.474	28.816	
Lattice 7	71.350	76.944	5.594		14.336	−0.072	14.408		2.118	0.077	2.041	
Lattice 8	61.359	−0.763	62.123		17.785	67.116	49.331		1.143	34.510	33.367	
2nd strategy												
Lattice 1	35.138	12.707	22.431	18.476	42.645	62.542	19.897	18.926	13.755	24.392	10.638	9.263
Lattice 2	47.527	57.088	9.561		21.503	4.106	17.397		8.988	4.646	4.342	
Lattice 3	28.650	29.877	1.226		52.309	77.738	25.429		18.775	29.877	11.102	
Lattice 4	38.641	38.019	0.622		35.758	24.999	10.759		14.793	13.045	1.749	
Lattice 5	64.862	49.150	15.713		18.462	19.016	0.554		3.126	7.370	4.243	
Lattice 6	52.473	7.153	45.320		25.252	71.796	46.544		4.658	27.718	23.060	
Lattice 7	71.350	53.194	18.155		14.336	17.750	3.414		2.118	6.110	3.992	
Lattice 8	61.359	26.582	34.777		17.785	45.202	27.417		1.143	16.120	14.977	

**Table A2:** Porosity, Elastic Modulus and Shear Modulus Mean Absolute Error (MAE) estimation for each training strategy.

Lattice nº	Simulated Relative Porosity (%)	Predicted Relative Porosity (%)	Relative Porosity AE	Relative Porosity MAE	Simulated Rel. Elastic Modulus E_Relative (%)	Predicted Rel. Elastic Modulus E_Relative (%)	Rel. Elastic Modulus E_Relative AE	Rel. Elastic Modulus E_Relative MAE	Simulated Rel. Shear Modulus G_Relative (%)	Predicted Rel. Shear Modulus G_Relative (%)	Rel. Shear Modulus G_Relative AE	Rel. Shear Modulus G_Relative MAE
3rd strategy												
Lattice 1	35.138	41.831	6.694	17.646	42.645	18.270	24.375	20.207	13.755	12.084	1.670	5.634
Lattice 2	47.527	91.267	43.740		21.503	−0.201	21.704		8.988	−0.187	9.175	
Lattice 3	28.650	32.668	4.018		52.309	26.848	25.461		18.775	14.455	4.320	
Lattice 4	38.641	89.796	51.155		35.758	−0.239	35.997		14.793	−0.160	14.954	
Lattice 5	64.862	60.900	3.963		18.462	−0.137	18.599		3.126	−0.101	3.227	
Lattice 6	52.473	32.554	19.919		25.252	22.132	3.120		4.658	12.956	8.298	
Lattice 7	71.350	66.871	4.479		14.336	−0.185	14.521		2.118	−0.143	2.261	
Lattice 8	61.359	68.560	7.201		17.785	−0.098	17.883		1.143	−0.025	1.167	
4th strategy												
Lattice 1	35.138	27.745	7.393	14.577	42.645	45.714	3.069	12.108	13.755	18.809	5.054	6.823
Lattice 2	47.527	86.467	38.940		21.503	0.292	21.211		8.988	−0.003	8.991	
Lattice 3	28.650	17.930	10.720		52.309	60.546	8.237		18.775	24.280	5.505	
Lattice 4	38.641	65.936	27.296		35.758	16.456	19.303		14.793	6.952	7.841	
Lattice 5	64.862	61.508	3.354		18.462	22.591	4.130		3.126	7.012	3.886	
Lattice 6	52.473	26.997	25.476		25.252	55.402	30.150		4.658	20.239	15.581	
Lattice 7	71.350	68.602	2.748		14.336	21.723	7.387		2.118	4.622	2.503	
Lattice 8	61.359	62.052	0.693		17.785	21.164	3.378		1.143	6.365	5.222	

**Table A3:** Porosity, Elastic Modulus and Shear Modulus Mean Absolute Error (MAE) estimation for each training strategy.

Lattice nº	Simulated Relative Porosity (%)	Predicted Relative Porosity (%)	Relative Porosity AE	Relative Porosity MAE	Simulated Rel. Elastic Modulus E_Relative (%)	Predicted Rel. Elastic Modulus E_Relative (%)	Rel. Elastic Modulus E_Relative AE	Rel. Elastic Modulus E_Relative MAE	Simulated Rel. Shear Modulus G_Relative (%)	Predicted Rel. Shear Modulus G_Relative (%)	Rel. Shear Modulus G_Relative AE	Rel. Shear Modulus G_Relative MAE
5th strategy												
Lattice 1	35.138	35.598	0.460	15.496	42.645	42.575	0.070	18.713	13.755	13.167	0.588	9.289
Lattice 2	47.527	47.341	0.187		21.503	29.543	8.040		8.988	17.428	8.440	
Lattice 3	28.650	33.927	5.276		52.309	43.521	8.789		18.775	14.347	4.428	
Lattice 4	38.641	31.366	7.275		35.758	43.336	7.578		14.793	22.453	7.660	
Lattice 5	64.862	44.621	20.242		18.462	35.414	16.952		3.126	7.862	4.736	
Lattice 6	52.473	21.655	30.818		25.252	67.866	42.614		4.658	25.450	20.793	
Lattice 7	71.350	47.203	24.147		14.336	36.286	21.950		2.118	7.237	5.118	
Lattice 8	61.359	25.800	35.559		17.785	61.499	43.714		1.143	23.696	22.553	
6th strategy												
Lattice 1	35.138	24.881	10.257	7.665	42.645	33.416	9.229	10.264	13.755	18.620	4.865	3.242
Lattice 2	47.527	57.971	10.444		21.503	18.641	2.862		8.988	7.243	1.745	
Lattice 3	28.650	24.990	3.660		52.309	33.358	18.951		18.775	18.512	0.263	
Lattice 4	38.641	36.310	2.331		35.758	26.531	9.227		14.793	14.152	0.641	
Lattice 5	64.862	62.012	2.850		18.462	5.179	13.283		3.126	3.943	0.817	
Lattice 6	52.473	34.515	17.957		25.252	25.039	0.213		4.658	13.551	8.893	
Lattice 7	71.350	79.106	7.756		14.336	3.789	10.547		2.118	0.402	1.717	
Lattice 8	61.359	67.421	6.062		17.785	−0.013	17.799		1.143	8.139	6.997	
